# Polyamine analog TBP inhibits proliferation of human K562 chronic myelogenous leukemia cells by induced apoptosis

**DOI:** 10.3892/ol.2014.2615

**Published:** 2014-10-13

**Authors:** QING WANG, YAN-LIN WANG, KAI WANG, JIAN-LIN YANG, CHUN-YU CAO

**Affiliations:** 1 Institute of Molecular Biology, Medical College, China Three Gorges University, Yichang, Hubei 443002, P.R. China; 2 College of Chemical and Pharmacy, Wuhan Engineering University, Wuhan, Hubei 430073, P.R. China

**Keywords:** tetrabutyl propanediamine, polyamine analog, chronic myeloid leukemia, apoptosis

## Abstract

The aim of the present study was to investigate the effects of the novel polyamine analog tetrabutyl propanediamine (TBP) on the growth of K562 chronic myelogenous leukemia (CML) cells and the underlying mechanism of these effects. MTT was used for the analysis of cell proliferation and flow cytometry was performed to analyze cell cycle distribution. DNA fragmentation analysis and Annexin V/propidium iodide double staining were used to identify apoptotic cells. The activity of the key enzymes in polyamine catabolism was detected using chemiluminescence. TBP can induce apoptosis and significantly inhibit K562 cell proliferation in a time- and dose-dependent manner. TBP treatment significantly induced the enzyme activity of spermine oxidase and acetylpolyamine oxidase in K562 cells, and also enhanced the inhibitory effect of the antitumor drug doxorubicin on K562 cell proliferation. As a novel polyamine analog, TBP significantly inhibited proliferation and induced apoptosis in K562 cells by upregulating the activity of the key enzymes in the polyamine catabolic pathways. TBP also increased the sensitivity of the K562 cells to the antitumor drug doxorubicin. These data indicate an important potential value of TBP for clinical therapy of human CML.

## Introduction

Chronic myelogenous leukemia (CML), one of the three most common forms of leukemia, is a hematopoietic stem cell disease which is mostly caused by abnormal expression of the oncoprotein BCR-ABL (1,2). At present, chemotherapy is the main clinical treatment strategy for CML. However, serious drug side-effects and multi-drug resistance caused by long-term chemotherapy reduce the efficacy of treatment (3,4). Hematopoietic stem cell transplantation is regarded as the most effective treatment for CML, but it is limited by numerous factors, including the availability of a donor, transplant rejection and the high expense (5). For these reasons, it is necessary to develop novel methods for CML therapy.

Polyamines, including spermidine, spermine and their diamine precursor putrescine, are polycationic alkylamines that are ubiquitously present in all mammalian cells. Polyamines are essential for eukaryotic cell growth, as these molecules participate in multi-physical processes, including gene transcription, regulation of protein function and cell membrane stability (6,7). Notably, tumor cells contain a higher concentration of polyamines than normal cells. The high level of polyamines promotes the proliferation, invasion and migration of tumor cells. Conversely, growth inhibition and apoptosis can be induced in tumor cells by reducing the polyamine concentration (8). Therefore, polyamine metabolism has been identified as an important novel target for antitumor therapy (9,10). Polyamine analogs are organic chemical compounds that are analogous to polyamines in molecular structure. Certain polyamine analogs can impose an inhibitory effect on tumor cells by disturbing polyamine metabolism. Tetrabutyl propanediamine (TBP) is a newly developed putricine analog developed by the present group. In our previous studies, TBP has been demonstrated to inhibit the growth and migration of human hepatocellular carcinoma HepG2 and osteosarcoma MG-63 cells by inducing apoptosis (11,12). In the present study, the effects of TBP on K562 cells and the underlying mechanism of these effects were further observed. The aim of the present study was to explore the potential value of TBP for the clinical therapy of human CML.

## Materials and methods

### Drugs and chemicals

The polyamine analog tetrabutyl propanediamine (TBP) was synthesized by Dr. Wang Kai (Wuhan Engineering University, Wuhan, China). Doxorubicin was produced by Zheijiang Hisun Pharmaceutical Co., Ltd. (Taizhou, China). RPMI-1640, Triton X-100, ribonuclease, propidium iodide (PI) and 3-(4,5-Dimethylthiazol)-2,5-diphenyltetrazolium bromide (MTT) were purchased from Sigma-Aldrich (St. Louis, MO, USA). Calf serum and the Annexin V kit were obtained from Invitrogen Life Technologies (Carlsbad, CA, USA). Agarose was purchased from Biowest LLC (Kansas, MO, USA).

### Cell culture

The K562 cells (China Center for Type Culture Collection, Wuhan, China) were grown in RPMI-1640 medium supplemented with 10% calf serum, 100 μg/ml streptomycin (North China Pharmaceutical Co., Ltd., Shijiazhuang, China) and 100 U/ml penicillin (North China Pharmaceutical Co., Ltd.) in a humidified 5% CO_2_ and 95% air incubator at 37°C.

### Assay of cellular proliferation

The K562 cells were seeded into 96-well culture plates [2,000 cells/well in 150 μl standard RPMI-1640 with the different concentrations (0, 12.5, 25,50 and 100 μmol/l) of TBP] in triplicate. After 24, 48 and 72 h of exposure to TBP, 50 μl MTT solution (250 μg/ml in RPMI-1640 medium) was added into each well and the cells were incubated at 37°C for 4 h. The plates were centrifuged at 380 × g for 5 min, the supernatant medium was removed and then 200 μl of dimethylsulfoxide was added to each well. After 20 min, the absorbance (A) of each well at 490 nm was recorded. The cell survival rate was calculated according to the following formula: Cell survival rate (%) = A_drug_ / A_control_ × 100.

### Cell cycle assay by flow cytometry

The K562 cells were treated with various concentrations of TBP for 24 h and harvested by centrifuging at 380 × g for 5 min. The cells were washed twice in ice-cold phosphate-buffered saline (PBS), suspended in PBS (containing 75% ethanol and 0.5 mmol/l EDTA) at 4°C for 30 min. The cells were then centrifuged to remove the supernatant and washed with PBS, resuspended in 500 μl PBS (containing 0.1% Triton X-100, 50 μg/ml ribonuclease and 0.1 mg/ml PI) at 4°C for 30 min in the dark and then assayed by flow cytometry.

### Detection of apoptosis by DNA fragmentation analysis

The K562 cells were treated with 50 and 100 μmol/l TBP for 24 h and harvested by centrifuging at 380 × g for 5 min. The cells were suspended in cell lysate buffer (50 mmol/l Tris-HCl pH 8.0, 20 mmol/l EDTA and 0.5% Triton X-100) and incubated on ice for 20 min. The lysate was centrifuged at 13,800 × g for 5 min to remove the nuclei. The supernatant was then harvested and extracted using an equal volume of phenol/chloroform. The DNA fragments in the extracted supernatant were precipitated by adding two volumes of 100% ethanol, 1/10 volume of 3 mol/l sodium acetate (pH 5.2) and centrifuging at 13,800 × g for 10 min. The pellets were suspended in 0.1X saline-sodium citrate buffer. Following treatment with deoxyribonuclease-free ribonuclease (10 mg/ml) for 30 min at 37°C, an equal volume of 2 mol/l NaCl was added and the solution was extracted again by phenol/chloroform and precipitated by two volumes of ethanol. The DNA pellets were then suspended in 30 μl double distilled water, separated by 1.5% agarose gel and visualized under UV illumination.

### Detection of apoptosis by flow cytometry following Annexin V/PI staining

The K562 cells were cultured in RPMI-1640 medium containing 0, 12.5, 25, 50 or 100 μmol/l TBP for 24 h and were harvested by centrifugation at 380 × g for 5 min. The cells were treated according to the manufacturer’s instructions, suspended in 500 μl PBS and then analyzed by flow cytometry.

### Determination of the activity of acetylpolyamine oxidase (APAO) and spermine oxidase (SMO)

The activity of APAO and SMO was detected by a modified chemiluminescent analysis (13). Briefly, the K562 cells were harvested, resuspended in 300 μl cell lysis buffer (glycine 83 mmol/l, Triton X-100 0.25%, pH 8.0) and stored at −80°C for 24 h to prepare the cell lysate. The enzyme activity in the cell lysate was then assayed in a 300-μl reaction, which consisted of 83 mmol/l glycine buffer (pH 8.0), 15 μmol/l luminol, 20 μg horseradish peroxidase, 0.2 mmol/l bromoethylamine (catalase inhibitor), 15 μmol/l deprenyl (copper containing amine inhibitor) and 0.15 mmol/l clorgyline (mitochondrial oxidase inhibitor), with 250 μmol/l N1-acetylspermine (for APAO activity) or 250 μmol/l spermine (for SMO activity) as substrates. All reagents, with the exception of the substrates, were combined and incubated for 2 min at 37°C, the tube was then transferred to the luminometer and the substrates were added. The resulting chemiluminescence was recoded over 20 sec. The enzyme activities were expressed as relative light units (RLU; RLU/μg protein/min).

### Statistical analysis

Data were presented as the mean ± standard deviation and were analyzed using a t-test performed using SPSS 17.0 software (SPSS Inc., Chicago, IL, USA). P<0.05 was considered to indicate a statistically significant difference.

## Results

### TBP inhibits the growth of K562 cells

**Figure 1:**
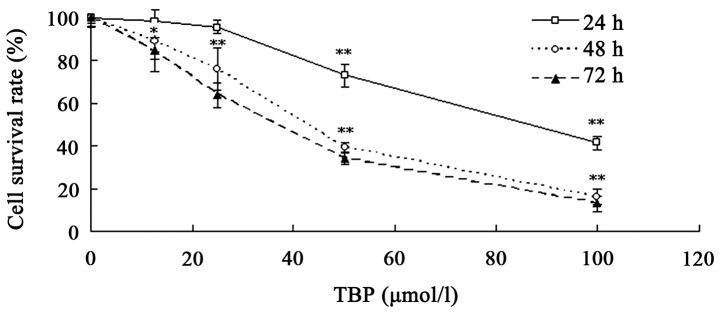
Inhibitory effect of TBP treatment on the proliferation of K562 cells. Each bar shows the mean ± standard deviation (n=3). ^*^P<0.05 and ^**^P<0.01, vs. 0 μmol/l TBP, as determined by the t-test. TBP, tetrabutyl propanediamine.

TBP has been demonstrated to inhibit the growth of solid tumor cell lines, including HepG2 and MG63 cells (11,12). The present study further investigated the effect of TBP on the proliferation of K562 cells using MTT assay. Various concentrations of TBP (range, 12.5–100 μmol/l) were used to treat K562 cells for 24, 48 or 72 h. The results revealed that TBP exerted a significant growth inhibition effect on K562 cells in a dose- and time-dependent manner. As shown in Fig. 1, the survival rates were 73.1, 39.4 and 34.1% when the cells were treated with 50 μmol/l TBP for 24, 48 and 72 h, respectively.

### TBP interferes with the cell cycle of K562 cells

**Figure 2:**
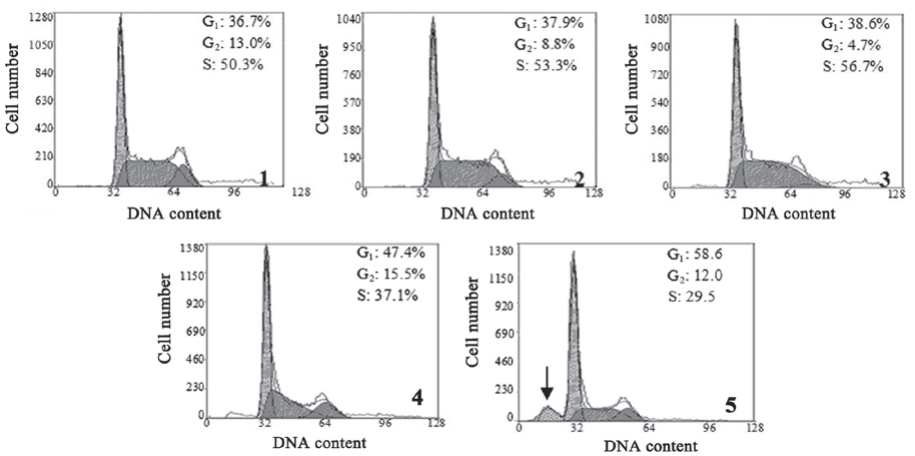
TBP interferes with the cell cycle of K562 cells. 1, K562 control cells; 2, K562 cells treated with 12.5 μmol/l TBP for 24 h; 3, K562 cells treated with 25 μmol/l TBP for 24 h; 4, K562 cells treated with 50 μmol/l TBP for 24 h; 5, K562 cells treated with 100 μmol/l TBP for 24 h. The arrow indicates the position of the sub-G_1_ apoptotic peak. TBP, tetrabutyl propanediamine.

The effect of TBP on the cell cycle of K562 cells was analyzed by flow cytometry. The results revealed that treatment with higher concentrations of TBP (50 and 100 μmol/l) resulted in a significantly increased cell ratio in the G_0_/G_1_ phase, but a decreased cell ratio in S phase. When the K562 cells were treated with 100 μmol/l TBP for 24 h, the sub-G_1_ apoptotic peak was visible. These results indicated that high concentrations of TBP can induce G_0_/G_1_-phase arrest and induce apoptosis in K562 cells (Fig. 2).

### TBP induces apoptosis of K562 cells

DNA fragmentation analysis and Annexin V/PI double staining were further used to identify TBP-induced apoptosis in the K562 cells.

**Figure 3:**
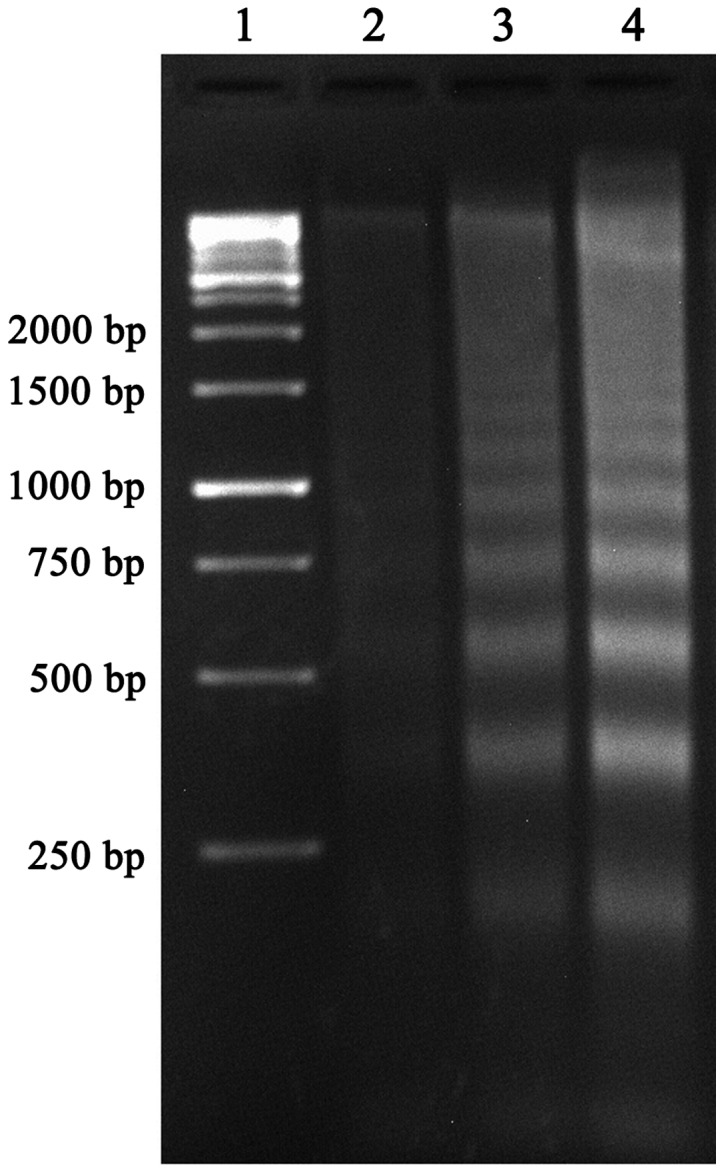
DNA fragmentation assay was used to determine TBP-induced apoptosis in K562 cells. Lane 1, DNA marker; lane 2, K562 control cells; lane 3, K562 cells treated with 50 μmol/l TBP for 24 h; lane 4, K562 cells treated with 100 μmol/l TBP for 24 h. TBP, tetrabutyl propanediamine.

As shown in Fig. 3, the clear DNA ladders that are a typical characteristic for apoptosis were observed in agarose gel electrophoresis subsequent to treating the K562 cells with TBP (50 and 100 μmol/l) for 24 h. The strength of the DNA ladders is dose-dependent.

**Figure 4:**
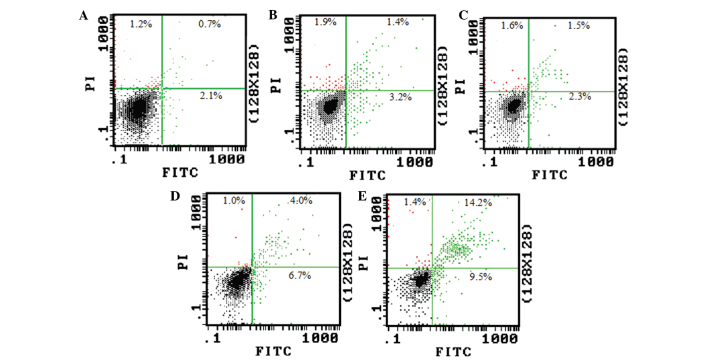
Annexin V/PI double staining was used to determine TBP-induced apoptosis in K562 cells. (A) Control cells; (B) cells treated with 12.5 μmol/l TBP for 24 h; (C) cells treated with 25 μmol/l TBP for 24 h; (D) cells treated with 50 μmol/l TBP for 24 h; (E) cells treated with 100 μmol/l TBP for 24 h. PI, propidium iodide; TBP, tetrabutyl propanediamine; FITC, fluorescein isothiocyanate.

The Annexin V/PI double staining and flow cytometry also revealed that the ratio of Annexin V/PI double-positive and Annexin V single-positive cells dramatically increased when the cells were treated with 50 and 100 μmol/l TBP (Fig. 4). This result is consistent with the DNA fragmentation assay.

### TBP upregulates the activity of SMO and APAO in K562 cells

**Figure 5:**
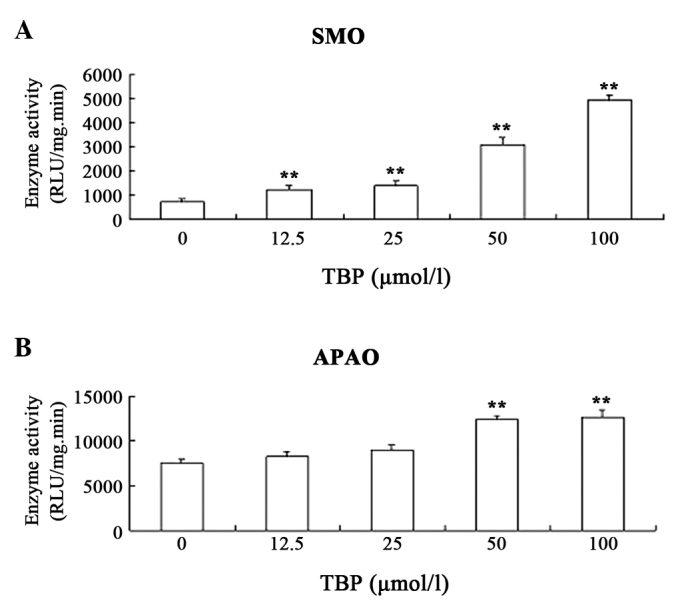
TBP upregulates the activity of SMO and APAO in K562 cells. The change in (A) SMO and (B) APAO activity in K562 cells treated with various concentrations of TBP for 24 h. Each bar exhibits the mean ± standard deviation (n=3), ^**^P<0.01, vs. 0 μmol/l TBP, as determined by the t-test. TBP, tetrabutyl propanediamine; SMO, spermine oxidase; APAO, acetylpolyamine oxidase.

The aforementioned results revealed that TBP inhibits K562 cell proliferation by inducing apoptosis. In order to explore the mechanism underlying these effects, the effect of TBP on the activity of SMO and APAO in K562 cells was investigated. SMO and APAO are important enzymes involved in polyamine catabolism. The enzymes catalyze degradation of polyamines and, at the same time, produce hydrogen peroxide (H_2_O_2_). SMO and APAO have each been proved to be important factors in inducing cell apoptosis (14). The results in the present study indicated that TBP upregulates the enzymatic activity of SMO and APAO in K562 cells. Notably, TBP could stimulate SMO activity much more strongly compared with APAO activity. Exposure of K562 cells to 50 μmol/l TBP for 24 h resulted in a ~4.5-fold induction of SMO activity and only a ~70% increase in APAO activity (Fig. 5).

### TBP improves the toxic response of K562 cells to doxorubicin

**Figure 6:**
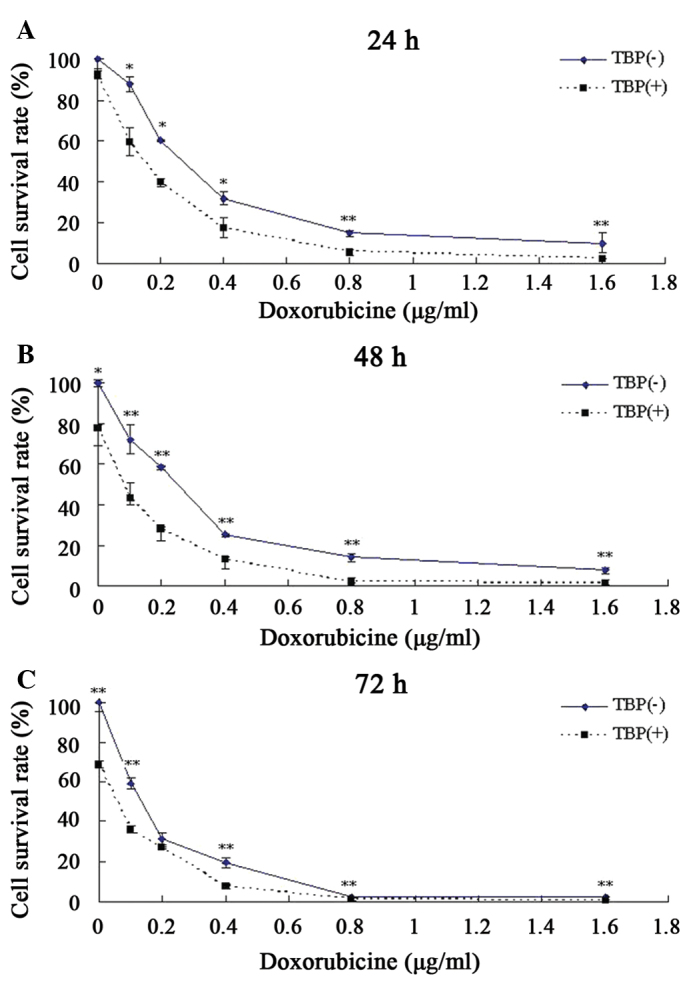
TBP enhances the toxic effects of doxorubicin on K562 cells. Doxorubicin was used, with or without 25 μmol/l TBP, to treat K562 cells for (A) 24 h, (B) 48 h and (C) 72 h. Each bar exhibits the mean ± standard deviation (n=3). ^*^P<0.05 and ^**^P<0.01, vs*.* TBP(−) groups of the same concentration and time, as determined by the t-test.

Doxorubicin is one of the first-line drugs in the clinical treatment of chronic myeloid leukemia, but long-term use of this drug can lead to drug resistance and reduce its therapeutic effect (15,16). One clinical strategy to reduce the drug resistance of tumor cells and enhance the antitumor effect is combination therapy using two or more drugs. The present study attempted to explore whether TBP can increase the sensitivity of K562 to doxorubicin when these two drugs are co-administered. According to the aforementioned data, although no evident growth inhibition was observed when K562 cells were treated with 25 μmol/l TBP, this same concentration of TBP could induce a ~2-fold increase in SMO activity. Therefore, this TBP concentration was used together with doxorubicin to co-treat K562 cells, and the results revealed that TBP significantly enhanced the toxic effect of doxorubicin on K562 cells. Compared with the K562 cells treated by doxorubicin only, TBP (25 μmol/l) co-treatment resulted in a 74% decrease in the required doses of doxorubicin for a 50% growth inhibition after 48 h (Fig. 6).

## Discussion

As a novel polyamine analog, TBP has been proved to inhibit the growth and migration of human hepatocellular carcinoma HepG2 cells and osteosarcoma MG-63 cells by inducing apoptosis (11,12). The present study further observed the effects of TBP on K562 cells and the underlying mechanism of these effects.

The present data revealed that TBP treatment significantly inhibited K562 cell proliferation and induced G_0_/G_1_-phase arrest in a time- and dose-dependent manner. In order to explore whether drug-induced apoptosis was the molecular mechanism underlying TBP-mediated growth inhibition, DNA fragmentation analysis and Annexin V/PI double staining were performed. The results revealed that, in the K562 cells treated with TBP, a DNA fragment ladder with the fragment lengths being multiples of 200 bp was observed on the agarose electrophoresis, which is a typical characteristic for apoptosis. Annexin V/PI and flow cytometry also revealed a significantly increased ratio of apoptotic cells following TBP treatment. These results demonstrated that TBP could inhibit the growth of K562 cells by inducing apoptosis.

Reactive oxygen species, including H_2_O_2_, are generally known as powerful inducers of apoptosis. Numerous studies have also demonstrated that polyamine depletion can trigger apoptosis in tumor cells. SMO and APAO are important enzymes involved in polyamine catabolism. These enzymes synergistically catalyze the degradation of spermine/spermidine and produce H_2_O_2_ as a side product (13,14). In order to evaluate the effect of TBP on SMO and APAO expression, the chemiluminescent assay was used to determine the enzymatic activity of SMO and APAO. The data revealed that the enzymatic activity of SMO and APAO in K562 cells were upregulated by TBP treatment. However, TBP imposed a more powerful induction on SMO compared with APAO. A low concentration of TBP (25 μmol/l) resulted in a ~2-fold induction in SMO enzymatic activity, but no evident induction for APAO activity was observed. A high concentration of TBP (100 μmol/l) can lead to a ~7-fold induction in SMO activity, but only ~1.7-fold induction for APAO activity. This result was consistent with the former results reported by Wang *et al,* who demonstrated that SMO was upregulated in response to exposure to the polyamine analog (17). These data suggest that the key mechanism underlying TBP induced-apoptosis in K562 cells may be associated with polyamine depletion and H_2_O_2_ accumulation caused by the powerful induction of SMO activity.

The present study also discovered that TBP at a low concentration (25 μmol/l) can significantly increase the sensitivity of the K562 cells to the antitumor drug doxorubicine and produce a synergistic antitumor effect, which means that in CML treatment, TBP could be used to increase the therapeutic efficacy and simultaneously reduce the side-effects and multi-drug resistance of doxorubicine by reducing the drug dosage.

In conclusion, the findings in the present study indicate that TBP can inhibit the growth and induce apoptosis in K562 cells by upregulating the key enzymes in polyamine catabolism and has a potential value for clinical therapy of human CML. Future studies are required, to clarify the effect of TBP in combination with other drugs on K562 cells and the effect of TBP on other leukemia cells.
